# Supplementary material for: Identification of the immune-associated enhancer RNA SATB1-AS1 as a novel biomarker for thymic cancer prognosis
Source: PLoS One. 2025 May 29;20(5):e0319965. doi: 10.1371/journal.pone.0319965 (PMC12122038; doi:10.1371/journal.pone.0319965)
Supplement: Attachment — Submitted filename: Review Wang et al.docx [file pone.0319965.s002.docx]

**Review of manuscript by Wang et al. “Identification of the immune-associated enhancer RNA SATB1-AS1 as a novel biomarker for thymic cancer prognosis”**

In the study by Wang et al., the authors performed a bioinformatics analysis using publicly available databases to explore the role of enhancer RNAs (eRNAs) as prognostic biomarkers in thymoma. The study successfully identified the immune-associated enhancer RNA SATB1-AS1 as a novel biomarker for thymic cancer prognosis.

This manuscript is well-written and easily understood. The introduction provides a clear and concise background, giving sufficient context for the study. The materials and methods section is well-structured and described in adequate detail. The manuscript can be accepted for publication after addressing the following comments.

**Specific Comments:**

1. **Data Extraction:**
   - Please specify the date when the data was extracted from the TCGA databases and Xena.
2. **Figure Quality and Assembly:**
   - Please provide details on how the figures were assembled.
   - The quality of the figures is low, making it difficult to distinguish details. Please improve the resolution of Figures 1, 3, and 4. If the issue is due to the PDF format of the manuscript, this comment can be disregarded.
3. **Discussion:**
   - There appears to be redundancy in the following sentence; please revise it for clarity: *"However, cancer treatment is limited by both intratumor and intertumor factors. However, cancer treatment is limited by intra- and intertumor heterogeneity, which requires the study of mechanisms that work across patient characteristics to develop innovative therapies."*
   - Please include references and elaborate on the known information about SATB1-AS1 and its connection to other known tumors.
4. **Conclusion:**
   - The following sentence appears incomplete; please revise for clarity and coherence: *(4) Differential expression of SATB1-AS1 across cancers and its impact on survival.*

After addressing these comments, the manuscript will be suitable for publication.
